# A tetravalent dengue virus-like particle vaccine induces high levels of neutralizing antibodies and reduces dengue replication in non-human primates

**DOI:** 10.1128/jvi.00239-24

**Published:** 2024-04-22

**Authors:** Daniel Thoresen, Kenta Matsuda, Akane Urakami, Mya Myat Ngwe Tun, Takushi Nomura, Meng Ling Moi, Yuri Watanabe, Momoko Ishikawa, Trang Thi Thu Hau, Hiroyuki Yamamoto, Yuriko Suzaki, Yasushi Ami, Jonathan F. Smith, Tetsuro Matano, Kouichi Morita, Wataru Akahata

**Affiliations:** 1 VLP Therapeutics, Inc., Gaithersburg, Maryland, USA; 2 Department of Tropical Viral Vaccine Development, Institute of Tropical Medicine, Nagasaki University, Nagasaki, Japan; 3 Department of Virology, Institute of Tropical Medicine, Nagasaki University, Nagasaki, Japan; 4 AIDS Research Center, National Institute of Infectious Diseases, Tokyo, Japan; 5 Joint Research Center for Human Retrovirus Infection, Kumamoto University, Kumamoto, Japan; 6 Department of Developmental Medical Sciences, Graduate School of Medicine, The University of Tokyo, Tokyo, Japan; 7 Management Department of Biosafety, Laboratory Animal, and Pathogen Bank, National Institute of Infectious Diseases, Tokyo, Japan; 8 Institute of Medical Science, University of Tokyo, Tokyo, Japan; 9 DEJIMA Infectious Disease Research Alliance, Nagasaki University, Nagasaki, Japan; Lerner Research Institute, Cleveland Clinic, Cleveland, Ohio, USA

**Keywords:** dengue, vaccine, VLP, NHP, challenge

## Abstract

Dengue virus (DENV) represents a significant global health burden, with 50% of the world’s population at risk of infection, and there is an urgent need for next-generation vaccines. Virus-like particle (VLP)-based vaccines, which mimic the antigenic structure of the virus but lack the viral genome, are an attractive approach. Here, we describe a dengue VLP (DENVLP) vaccine which generates a neutralizing antibody response against all four DENV serotypes in 100% of immunized non-human primates for up to 1 year. Additionally, DENVLP vaccination produced no ADE response against any of four DENV serotypes *in vitro*. DENVLP vaccination reduces viral replication in a non-human primate challenge model. We also show that transfer of purified IgG from immunized monkeys into immunodeficient mice protects against subsequent lethal DENV challenge, indicating a humoral mechanism of protection. These results indicate that this DENVLP vaccine is immunogenic and can be considered for clinical evaluation. Immunization of non-human primates with a tetravalent DENVLP vaccine induces high levels of neutralizing antibodies and reduces the severity of infection for all four dengue serotypes.

**IMPORTANCE:**

Dengue is a viral disease that infects nearly 400 million people worldwide and causes dengue hemorrhagic fever, which is responsible for 10,000 deaths each year. Currently, there is no therapeutic drug licensed to treat dengue infection, which makes the development of an effective vaccine essential. Virus-like particles (VLPs) are a safe and highly immunogenic platform that can be used in young children, immunocompromised individuals, as well as healthy adults. In this study, we describe the development of a dengue VLP vaccine and demonstrate that it induces a robust immune response against the dengue virus for over 1 year in monkeys. The immunity induced by this vaccine reduced live dengue infection in both murine and non-human primate models. These results indicate that our dengue VLP vaccine is a promising vaccine candidate.

## INTRODUCTION

Dengue is a mosquito-borne viral disease that infects nearly 400 million people worldwide each year, and in severe cases causes dengue hemorrhagic fever (DHF), which is responsible for approximately 10,000 deaths each year (1, 2). Dengue virus (DENV) is spread by both *Aedes aegypti* and *Aedes albopictus* mosquitos, which are endemic to more than 100 countries in all 6 habitable continents (3, 4). Furthermore, in the coming decades, the range of both vector hosts will likely increase due to climate change, further increasing the population at risk of infection (5). No therapeutic drug to treat dengue infection is currently licensed, which makes an effective vaccine essential to meeting this global health threat.

A significant obstacle in the development of a dengue vaccine is the phenomenon known as antibody-dependent enhancement (ADE) (6). There are four closely related, but antigenically distinct, DENV serotypes (DENV1-4). An individual’s first DENV infection is typically mild or asymptomatic. However, a subsequent infection with a different DENV serotype may result in more severe disease due to ADE. ADE occurs when pre-existing, but non-neutralizing, anti-DENV antibodies against one DENV serotype form a DENV-antibody immunocomplex which enhances cellular entry into Fc-receptor-bearing cells and worsens the infection rather than preventing it (7). Though no clear clinical evidence has emerged yet, there is concern that an imbalanced vaccine could generate ADE, causing a person’s first dengue exposure to lead to more severe illness. Therefore, to prevent ADE risk, any dengue vaccine must produce strong neutralizing antibody (NAb) responses against all four serotypes simultaneously.

To date, two dengue vaccines have been licensed and approved. The first one, Dengvaxia, is a live-attenuated tetravalent vaccine developed by Sanofi Pasteur and approved in 2016. However, long-term safety and efficacy studies revealed variable efficacy based on age and dengue sero-status at the time of vaccination, as children without prior dengue exposure at initial vaccination exhibited an elevated risk of severe dengue infection and hospitalization relative to children in the control group (8, 9). Because of this risk, Dengvaxia is only recommended for persons 9–16 years of age in the areas of high dengue-endemicity (>70% seroprevalence) (10, 11). The second approved vaccine, Qdenga (TAK-003), is a live-attenuated tetravalent vaccine developed by Takeda. Because of the lack of data to assess the risk of enhanced disease in seronegative vaccinated children following DENV3 and 4 infection, the Strategic Advisory Group of Experts on Immunization (SAGE) strongly recommends further post-marketing studies to determine efficacy-risk profile in seronegative persons and recommended use of Qdenga only for children ages 6–16 in areas of high dengue disease burden (12). Therefore, developing an advanced dengue vaccine remains a major global health objective.

Virus-like particles (VLPs) are self-assembling structures composed of viral structural proteins without genomic DNA or RNA. VLP vaccines present a repetitive, high-density antigen profile that closely mimics the morphology of an authentic virus, making them highly immunogenic (13). However, the absence of the genomic information required for replication makes them relatively safe even in young children and immunocompromised individuals. The ability to immunize young children has critical importance for reducing dengue morbidity as a study of DHF prevalence in Thailand demonstrated that infants < 1 year old represent a significant proportion of severe DHF cases (14). One hypothesis for the cause of severe dengue infection in infants is that it stems from ADE caused by maternal DENV antibodies acquired *in utero*. As the passively transferred maternal antibody titer wanes, the once protective antibody can result in enhanced dengue infection in the infant (15, 16). While infants can receive live vaccines, there is concern for a live-attenuated dengue vaccine that the presence of the maternal antibodies may negatively affect the replication of one or more attenuated serotypes, leading to an imbalanced response (17, 18). Immunocompromised individuals are another group at high risk of severe dengue infection (19); however, live-attenuated vaccines are not recommended due to the risk of increased replication or genetic reversion (20, 21). VLP vaccines, on the other hand, are safe for immunocompromised persons because they lack the genetic material required for replication and reversion. These characteristics make a VLP vaccine attractive for protecting both high-risk groups.

In this study, we describe a tetravalent DENVLP vaccine and demonstrate that it induces a durable NAb response against all four DENV serotypes for up to 1 year in non-human primates (NHPs). Furthermore, *in vitro* ADE activity was not detected against any serotype throughout the 1-year timeframe. We also show that the anti-DENV neutralizing antibodies induced by this vaccine are capable of reducing viral replication in rodents and NHPs.

## RESULTS

### A tetravalent DENV VLP vaccine generates robust and long-lasting immunity

**FIG 1:**
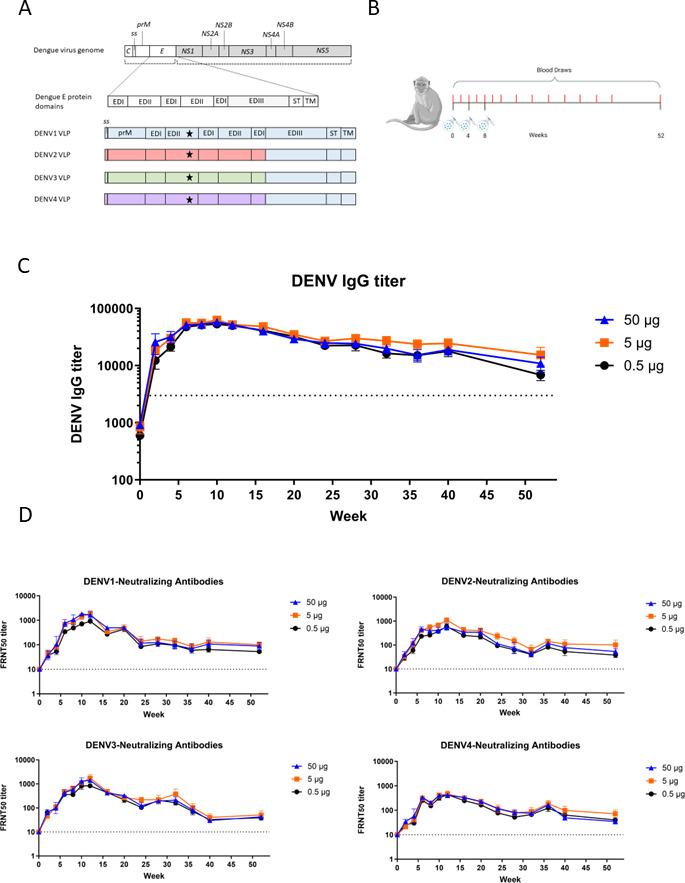
A Tetravalent DENVLP vaccine generates robust antibody response. (**A**) Schematic illustration of the DENV genome and DENV1-4 VLP expression vectors. C, capsid; ss, signal sequence; prM, precursor membrane; E, envelope; NS1-5, Non-structural proteins 1–5; EDI–III, envelope domains I–III; ST, stem; TM, transmembrane domain. Star symbol indicates location of F108A substitution in VLP expression constructs. (**B**) Graphic of macaque immunization experiment. Tetravalent DENVLP (0.5, 5 and 50 µg, *n* = 6 per dose group) adjuvanted with alum was administered at weeks 0, 4 and 8. Serum samples (shown in red) were taken every 2 weeks from weeks 0 to 12 and every 4 weeks from weeks 12 to 52. (**C**) The mean anti-DENV IgG titer for all three dose groups was determined by ELISA for each time point. Serum samples from each macaque and timepoint were diluted 1:1,000, and after labeling with a horseradish peroxidase (HRP)-conjugated secondary antibody and developed with substrate, the OD_492_ value for each sample was determined from a standard curve. The minimum threshold for a positive result, shown as the dotted line, is set to an IgG titer of 3,000. The statistical difference in mean IgG titer among the three dose groups at each timepoint was measured using a one-way ANOVA with post-hoc Tukey’s multiple comparison tests, and the greatest difference between any two dose groups was plotted above each timepoint (unlabeled = *P* > 0.05). (**D**) The mean NAb titer against all four DENV serotypes for each dose group and time point as determined by FRNT_50_ assay. Serum samples from each macaque and timepoint were serially diluted and incubated with DENV serotypes 1–4 prior to inoculation of Vero cells. The endpoint dilution capable of neutralizing half of the viral foci relative to Vero cells incubated with DENV1-4 alone was recorded for each macaque and timepoint was recorded, and the mean value for each dose group and sample timepoint was plotted. The statistical difference in FRNT50 titer among the three dose groups at each timepoint was measured using a one-way ANOVA with post-hoc Tukey’s multiple comparison tests, and the greatest difference between any two dose groups was plotted above each timepoint (unlabeled = *P* > 0.05).

We have previously described the generation of an envelope-modified tetravalent DENVLP vaccine (22). The prM-E region of DENV1-4 was selected for VLPs due to its ability to spontaneously form the VLP structure as well as the ability to generate strong NAb levels. In all four constructs, a mutation in the fusion loop (F108A) of envelope domain II (EDII) enabled a significant increase in VLP production without disrupting the vaccine’s ability to induce neutralizing antibodies (Fig. 1A). In the DENVLP2, 3, and 4 constructs, the envelope domain III, stem, and transmembrane anchor (EDIII/ST/TM) of the native serotype were replaced with the EDIII/ST/TM of DENV1 (shown in blue) in order to increase vaccine production (22). Before the immunization experiments conducted here, we confirmed via electron microscope (EM) imaging that all four DENVLPs resembled the structures of other flavivirus VLPs (Fig. S1A through D).

To approximate the human immune response to DENVLP vaccination, we conducted immunogenicity and protection studies in NHPs, which were previously used to demonstrate preclinical immunogenicity for the approved dengue vaccines prior to their use in humans (17, 18). Cynomolgus macaques (*Macaca fascicularis*) were immunized with one of three doses (0.5, 5, or 50 µg total DENVLP, containing equal amounts of each serotype, *n* = 6 per group) of tetravalent DENVLP vaccine at 0, 4, and 8 weeks (Fig. 1B). The total IgG antibody titer against all four DENV serotypes was measured via enzyme-linked immunosorbent assay (ELISA) (Fig. 1C), and the mean anti-DENV IgG titer at the peak (week 10) for the lowest vaccine dose (0.5 µg) was 53,350 ± 4,977, which was not significantly different from the mean IgG titer of 57,510 ± 14,314 for the highest dose (50 µg). By 52 weeks after the first immunization, the total anti-DENV IgG titer had declined to between 1/4th and 1/8th of the peak IgG titer observed depending on the dose of vaccine delivered, but the IgG titer values for the highest and lowest doses of vaccine were still not statistically significant. The IgG titer for all three vaccine doses remained above the threshold of detection at the 52-week point, indicating that this vaccine regimen produced a humoral response up to 1 year and beyond. The statistical difference among all three dose groups at each timepoint was measured by one-way ANOVA, and no statistically significant difference between any dose group was measured.

To determine whether the antibodies produced by the DENVLP vaccine could sufficiently neutralize all four dengue serotypes, we measured the NAb titer against each specific virus serotype (DENV1-4) in the serum of the same macaques. We observed substantial NAb titers generated to each DENV serotype, with peak NAb titers observed at either week 10 or 12 (1,848, 1,120, 1,732, and 462 against DENV1-4, respectively) for all vaccine doses against all four serotypes (Fig. 1D). By week 52, the NAb titers had waned from the peak (100, 102, 51, and 72 against DENV1-4, respectively), but remained well above the threshold of detection of 10, indicating that a NAb response was maintained for up to 1 year or beyond following immunization. At all timepoints the differences in NAb titer among the three dose groups were not statistically significant as determined by one-way ANOVA, suggesting that all three dose levels could produce a robust neutralizing response. Comparing the NAb titer among the four strains for all 18 immunized animals, the greatest fold difference in NAb titer observed was a 5-fold difference between DENV1 and DENV4 at week 8; however, following the third vaccine dose the serotypes with the highest and lowest NAb titers varied from sample to sample (Fig. S2), suggesting a relatively balanced response to each serotype.

### Tetravalent DENVLP vaccination does not generate *in vitro* ADE for DENV serotypes 1–4

**FIG 2:**
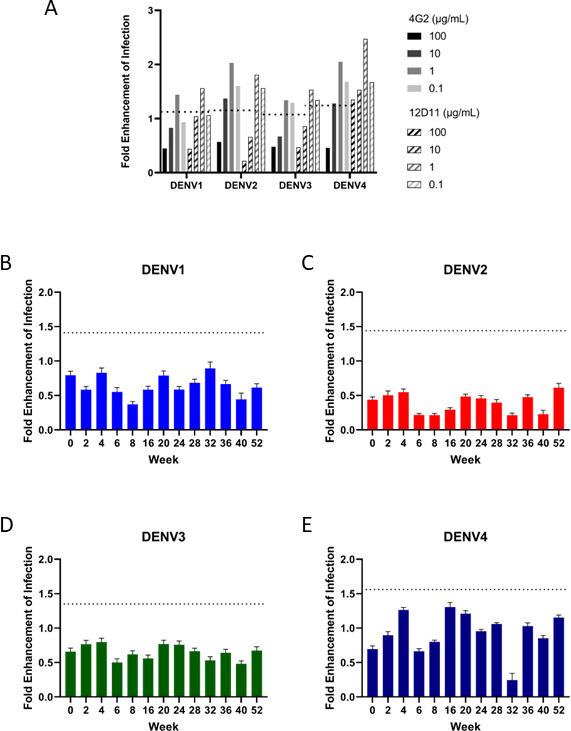
Dilution of neutralizing antibodies can cause ADE activity, but not for macaque sera diluted 1:10. (**A–E**) DENV1-4 were incubated with monoclonal antibodies at indicated dilutions (**A**) or sera from immunized macaques (*n* = 18) diluted 1:10 (**B–E**) prior to inoculation of FCγR-BHK cells. After 2 days, infected cells were counted, and the ratio of infected cells in serum- or antibody-incubated wells to infected cells in wells inoculated with virus alone was plotted as the fold enhancement of infection. The mean value plus three SDs for three negative control wells was used as the threshold for enhancement (shown as a dotted line in A–E).

Immunization with dengue vaccines can potentially cause ADE by inducing both neutralizing and non-neutralizing antibodies. When the level of neutralizing antibodies is insufficient to fully neutralize virions, infection can be enhanced rather than suppressed. To examine whether the antibodies generated by DENVLP immunization of cynomolgus monkeys were capable of increasing infectivity via ADE, DENV1-4 virions were incubated with the indicated dilutions of either purified monoclonal neutralizing antibodies (Control) or serum from tetravalent DENVLP-immunized macaques, and then Fc-gamma receptor-expressing baby hamster kidney cells (BHK-FcγR) were infected with the antibody-virion complexes. With two purified monoclonal anti-dengue neutralizing antibodies, high levels of antibody suppressed the infection of BHK cells, resulting in a ratio of infectivity <1 when compared to virus-infected cells alone (Fig. 2A). However, as the monoclonal antibodies are diluted further, the ratio of infectivity rises above the indicated threshold for ADE, as measured by the plaque counts for virus alone +3 standard deviation (SD). This demonstrates that dengue infection of the BHK-FcγR cells will be enhanced as antibody titer decreases. For DENVLP-immunized cynomolgus monkeys (*n* = 18), a 1:10 serum dilution failed to generate ADE through 52 weeks (Fig. 2B through E), with levels consistently below the acceptable threshold for ADE.

Further dilution of the immunized macaque sera to 1:100 and 1:1,000 also failed to induce ADE for any of the four serotypes (Fig. S2A and B). The only exception to this was the sera from week 16, which appeared to cause ADE with DENV4 at both 1:100 and 1:1,000 dilutions. While concerning, the absence of continued ADE at later timepoints suggested that this was not necessarily a consistent effect of the vaccine.

### DENVLP vaccination reduces viral replication

**FIG 3:**
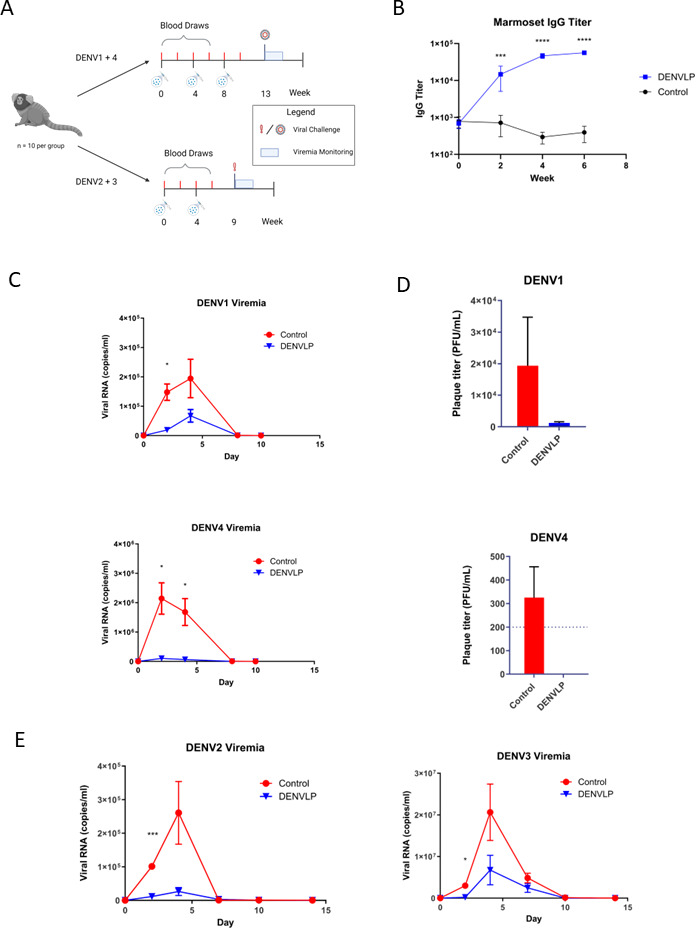
DENVLP-immunized marmosets are protected from severe viremia upon DENV1-4 challenges. (A) Graphic of marmoset challenge experiment. Forty marmosets were divided into four equal groups and immunized with either 5 µg tetravalent DENVLP (*n* = 6) or vehicle control (*n* = 4) either twice (weeks 0 and 4, DENV2 and 3 challenge) or three times (weeks 0, 4 and 8, DENV1 and 4 challenge). (B) Serum samples drawn from DENVLP- or control-immunized marmoset from weeks 0 to 6 were used to measure the total anti-DENV IgG titer via ELISA. The statistical difference in mean IgG titer between the two conditions at each timepoint was measured using an unpaired *t*-test with Welch’s correction (***P < 0.001, ****P < 0.0001). (C and E) Measurement of blood viremia by RT-qPCR. Viral RNA was purified from marmoset blood samples drawn every 2 days from day 0 to 10 (DENV1+4) or day 0 to 14 (DENV2+3), and then measuring the total number of genomic copies per mL of blood using serotype-specific primers against DENV E protein. The statistical difference in mean Viral RNA copies/mL between immunized and control marmosets at each timepoint was measured using an unpaired t test with Welch’s correction (*P < 0.05, ***P < 0.001, unlabeled = *P* > 0.05). (**D**) Measurement of blood viremia by plaque assay. Blood samples drawn from each marmoset at day 4, the peak of mean viremia (measured by RT-qPCR), were serially diluted and incubated with Vero cells, and the mean number of PFU per mL of blood was measured for each marmoset.

Next, we sought to determine the protective effect of the DENVLP vaccine against a live DENV challenge. Marmosets (*Callithrix jacchus*) were selected for the live challenge studies because they develop high levels of viremia upon primary DENV infection. Marmosets were immunized either twice (in DENV2 and 3 challenges) or three times (in DENV1 and 4 challenges) with 1.25 µg per serotype of all four DENVLPs or with vehicle control, then challenged 5 weeks after the final immunization with one of the four DENV serotypes (1 × 10^5^ plaque-forming units (PFU) each) (Fig. 3A). The challenge strains selected differed in amino acid sequence of the E protein from the VLP constructs by 1%–3% (data not shown). All the immunized marmosets elicited robust antibody responses against DENV as measured by ELISA (Fig. 3B). All control marmosets challenged with DENV1 or DENV4 developed viremia, with peak viral RNA observed at 2–4 days post-challenge via RT-qPCR (Fig. 3C). In contrast, all DENVLP-immunized marmosets showed significantly reduced viral RNA levels at peak infection (67,000 copies/mL for DENVLP-immunized animals vs 194,000 copies/mL for controls) (Fig. 3C). When total infectious virions were measured via plaque assay, control marmosets challenged with DENV1 showed peak viremia around 1.9 × 10^4^ PFU/mL at day 4, while DENVLP-immunized marmosets had only 1.2 × 10^3^ PFU/mL (Fig. 3D), a more than 10-fold decline in infectious virions. Similarly, the control marmosets challenged with DENV4 developed peak viremia more than 3 × 10^2^ PFU/mL, whereas VLP-immunized marmosets had no detectable plaques observed at day 4 (Fig. 3D). Upon challenge with DENV2 and DENV3, we failed to detect infectious virions in either immunized or control samples via plaque assay (data not shown). However, a significant increase in DENV2 and DENV3 genomic RNA copies/mL was observed in the blood of control animals via qPCR, suggesting that viral replication was occurring in unvaccinated marmosets (Fig. 3D). At the peak of viral RNA load in the control group, the level of DENV2 viral RNA observed in VLP-immunized marmosets was nearly 10-fold lower (2.6 × 10^4^ copies/mL for DENVLP-immunized vs 2.6 × 10^5^ copies/mL for controls) and the level of DENV3 viral RNA observed in DENVLP-immunized marmosets was more than 3-fold lower (6.8 × 10^6^ copies/mL for DENVLP-immunized vs 2.1 × 10^7^ copies/mL for controls) (Fig. 3E).

**FIG 4:**
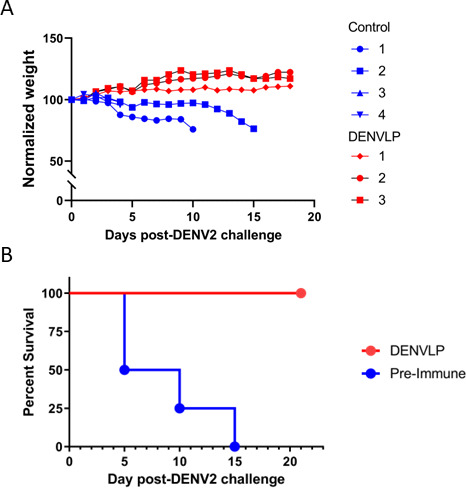
Passive transfer of DENVLP-immunized macaque sera prevents lethal DENV2 infection of AG129 mice. Total IgG was purified from blood samples drawn from all 18 macaques either prior to first immunization (pre-immune, *n* = 4) or at week 12 (DENVLP, *n* = 3), and the purified IgG was then introduced into AG129 mice via i.p injection 24 h prior to challenge with a lethal dose of DENV2. (**A**) The weight of each individual mouse following DENV2 challenge normalized to its initial pre-challenge weight. (**B**) The overall survival rate of AG129 mice receiving IgG from macaques before or after DENVLP immunization.

To determine whether the protection against viral infection generated by the DENVLP vaccine was conferred primarily by the antibodies produced, we investigated whether IgG from DENVLP-immunized macaques could protect against lethal challenge in a passive transfer animal model (23). Purified total IgG from macaque sera drawn at either week 0 (pre-immune) or week 12 (VLP-immunized) was transferred into AG129 mice (*n* = 4 per group), which lack interferon receptors. The mice were subsequently challenged with a lethal dose (1 × 10^6.5^ PFU) of DENV2 24 h after IgG transfer. While mice that received pre-immune IgG developed severe infection and died by 15 days post-infection, mice that received DENVLP-immunized IgG were protected from lethal infection (Fig. 4). These results indicate that the humoral immune response induced by tetravalent DENVLP immunization confers protection against severe dengue infection in mice.

## DISCUSSION

To meet the growing global health challenge of dengue, a next-generation vaccine must meet several key criteria to be effective: it must be able to be used in high-risk groups, quickly generate protective immunity against all four dengue serotypes, and ensure that protection is durable over the long term. Here, DENVLP-immunized macaques generated a robust Nab titer against all four dengue serotypes within just 2 weeks of immunization, with a peak Nab titer observed at 4 weeks after the second booster immunization. The induction of a high Nab titer is essential to avoid a “danger zone” where anti-DENV antibodies are present at lower levels than what is required to neutralize the virions, which could leave vaccine recipients at risk of ADE until subsequent booster doses raise their Nab titers (24). Another risk period could occur later after vaccination if the NAb titer gradually wanes into dangerous non-neutralizing levels. The immunization of macaques with DENVLP vaccine generated a relatively balanced NAb titer against all four serotypes from 2 to 52 weeks. Additionally, the anti-dengue antibodies present in macaque sera from week 2 to 52 failed to induce ADE for any other 4 dengue serotypes using a BHK-FcγR reporter system. These results suggest that the DENVLP vaccine is promising, but additional studies would be necessary to confirm the absence of ADE. Both the speed and durability of the immunogenicity generated in macaques provide evidence that the DENVLP vaccine shows promise as a next-generation dengue vaccine.

All three doses of DENVLP vaccine (0.5, 5 and 50 µg total VLP) produced robust peak Nab titers in macaques, and at all timepoints there was no statistically significant difference between the NAb titer induced by the highest and lowest vaccine doses in any serotype. These results suggest that the quantity of these DENV antigens required to induce a robust immune response may be lower than even the lowest dose delivered here. For comparison, immunization of rhesus macaques with a recombinant subunit vaccine produced much lower NAb titers even when vaccinating with 250 µg total DENV E protein (25). A plausible explanation for the efficient immunization with low doses of the DENVLP vaccine is that the highly structured, repetitive structure of VLPs allows for the robust stimulation of T- and B-cells required for a strong immune response. For this reason, VLP vaccines may be an attractive alternative to other non-live vaccination methods. However, a dose variation clinical trial will need to be conducted to establish the minimum dose of VLP vaccine required to induce durable protective immunity in humans.

Different neutralization assays have inherent variation, but there is nonetheless value in comparing the NAb titers for each serotype produced by tetravalent DENVLP vaccination in NHPs to the NAb titers produced by other vaccines. Prior to their use in humans, both currently approved live attenuated vaccines were first tested in NHPs. Immunization of macaques with equal doses of each serotype of the Dengvaxia vaccine produced a robust DENV4 NAb response at 3 months after immunization, but a 10-fold lower geometric mean titer (GMT) of DENV1 NAbs (26). This mirrored the overall efficacy of Dengvaxia in seronegative children in phase III studies, where the risk ratio for hospitalization with DENV4 was lower than that of DENV1, 2 or 3 (27). Similarly, immunization of macaques with a tetravalent Qdenga formulation produced a DENV2 NAb GMT that was 5-fold higher than DENV1 (formulation 2) or 15-fold higher than DENV4 NAb GMT (formulation 3) (28). This mirrored the overall efficacy of Qdenga vaccination in seronegative recipients, where they showed good efficacy against DENV2 and poor efficacy against DENV3 and 4 (29). Following DENVLP immunization, all four serotypes showed consistent and comparable NAb titer across weeks 2–52. The NAb titers against all four DENV serotypes rose and then declined at a similar rate, indicating that the circulating antibodies were relatively balanced. The largest difference in NAb titer observed at any one timepoint was a five-fold difference between DENV1 and DENV4 at week 8. The balanced NAb titers throughout the 1-year period likely contributes to the absence of *in vitro* ADE activity that we observed. We hypothesize that both the balanced NAb titers and the absence of ADE stem from the consistent presentation of equal quantities of antigen for DENV1-4 by the VLP vaccine system. We are hopeful that these results would predict protection against all four strains of dengue, but this would have to be tested directly against currently circulating strains.

Because of the high and consistent NAb titer generated by the DENVLP vaccine, we were able to demonstrate that this vaccine can reduce viral replication in marmosets following live dengue infection with all four serotypes, and the antibodies generated prevent lethal infection in a passive-transfer model. Marmosets are susceptible to infection by all four DENV serotypes, however vaccination with the DENVLP vaccine provided protection against viremia, as observed by both plaque assay and qPCR. The passive transfer of purified IgG from VLP-immunized macaques to immunocompromised AG129 mice provided protection against DENV2 infection, confirming that the antibodies induced by vaccination were responsible for this protection.

DENV produces many immature particles during infection, and antibodies against the immature prM protein have previously been implicated as a risk factor for ADE and severe secondary infection (30). In accordance with this risk, there may be some concern that a vaccine containing the uncleaved prM protein (instead of mature M protein) may increase ADE susceptibility. However, our measures of *in vitro* ADE activity using FcγR-expressing BHK cells indicates that this is not the case for any of the four DENVLP constructs tested. The serotype-specific immunity is presumably conferred by the E proteins for each serotype, and these results suggest that the neutralizing antibodies generated against the E protein are sufficient to counteract any prM antibodies that may enhance infection. Another previously reported risk factor for ADE is antibodies specific to the fusion loop region of the E protein (31). It is possible that in these DENVLP constructs, which contain the F108A mutation to improve overall yield, may also limit ADE by this mechanism.

While these results are promising, there are several limiting factors that qualify our interpretation of the results. The BHK-FcγR *in vitro* system can indicate whether the virions taken up via Fcγ receptors have been insufficiently neutralized; however, studies measuring the correlation of this assay with ADE or severe disease in humans are ongoing (32). Similarly, while marmosets are an attractive model for DENV infection, the protection conferred by the DENVLP vaccine will need to be confirmed in a clinical trial in humans. Ultimately, these results demonstrate that a VLP-type vaccine is a promising candidate for inducing balanced, robust immunity against all four dengue serotypes, and the results support further evaluation of the safety and efficacy of these constructs in human clinical trials.

## MATERIALS AND METHODS

### DENVLP preparation

The construction of DENVLP1-4 expression plasmids and production of VLPs has been reported previously (22). Briefly, Freestyle 293 F cells (Thermo Fisher Scientific) cultured in FreeStyle 293 Expression Medium (Thermo Fisher Scientific) were transfected with DENV VLP-expressing plasmids. The culture supernatant at 4 days after transfection was clarified, concentrated, and purified by HiTrap Q XL (GE Healthcare Life Sciences) and Foresight CHT Type II (Bio-Rad) columns with sodium phosphate gradient. Total protein concentration of purified DENV VLP was measured by Quick Start Bradford Protein Assay (Bio-Rad) with a final yield of up to 10 mg/L for all four serotypes. Purity of the DENV VLP was assessed by SDS-PAGE followed by Coomassie dye-based staining using QC colloidal Coomassie stain (Bio-Rad). The morphologies of DENV VLPs were analyzed at the National Institute of Infectious Disease in Japan (NIID) Microscope Facility to confirm consistency prior to their use in animal studies.

### Animal studies

All cynomolgus monkeys and common marmosets were purchased from Hamri Co. Ltd. (Koga, Ibaraki, Japan) and CLEA Japan, Inc. (Tokyo, Japan), respectively and housed at NIID facilities. All animal procedures were conducted in accordance with the NIID “Guides for Animal Experiments Performed at National Institute of Infectious Diseases”, approved by the Animal Welfare and Animal Care Committee of NIID, Tokyo, Japan. Sedation of animals for vaccination, infection, and blood sampling was performed with intramuscular ketamine hydrochloride (50 mg kg^−1^) and xylazine (3 mg kg^−1^).

For immunogenicity studies, 18 healthy, 4 years old male cynomolgus monkeys were used. Prior to initiation of the study, serum anti-flavi IgG titer was assessed by ELISA to confirm that they had not previously infected with flaviviruses. VLP was mixed with Alum adjuvant immediately before intramuscular injection to femoral region at weeks 0, 4, and 8. Blood samples were taken every 2 weeks during the first 12 weeks, and at every 4 weeks from weeks 12 to 52. Sera samples were heat-inactivated by incubating at 60 °C for 30 min. Measurement of serum anti-DENV IgG titer, neutralization antibody (NAb) titer and ADE was conducted as described below.

For the DENV viremia protection studies, 40 1–2-years old common marmosets (equally divided males and females) were divided into 4 challenge groups. Each challenge group was then further divided, with six marmosets receiving 5 µg total tetravalent DENVLP formulated with Alum adjuvant (DENVLP group) and four marmosets receiving vehicle solution (5% sucrose/5 mM sodium phosphate, pH 7.2) as a control. The marmosets receiving DENV1 or DENV4 challenge strains received three total immunizations at weeks 0, 4 and 8, while marmosets receiving DENV2 or DENV3 challenged were immunized twice at weeks 0, and 4. For all marmosets, serum samples were taken every 2 weeks until DENV challenge for measurement of serum anti-DENV IgG titer. At 5 weeks after final immunization (week 9 for DENV2/3, week 13 for DENV1/4) all marmosets were challenged with 1 × 10^5^ PFU DENV (DENV1: 01–44 strain, DENV2: DHF0663 strain, DENV3: DSS1403 strain, DENV4: 05–04 strain) via subcutaneous injection in the back (divided between 2 and 3 inoculation sites). Following DENV challenge, blood was taken every 2 days from day 0 to day 10 (DENV1/4) or day 14 (DENV2/3) for measurement of viremia using RT-qPCR and plaque assays as described below.

### Immunogenicity assays (ELISA)

ELISA was performed as previously described (22). Briefly, 96-well ELISA plates were coated with purified Japanese encephalitis virus (JEV, strain: JaOArS982) or DENV mixed antigens at 250 ng/well at 4 °C overnight. The plates were then blocked with undiluted BlockAce (DS pharma Biomedical) for 1 h at room temperature. Primary probing of the antigen was conducted using sera samples, and standard control was diluted at 1:1,000. 1:1,000 diluted HRP conjugated anti-mouse IgG antibody (American Qualex) was added. Serum IgG titers were calculated from standard curve as described previously (33). A sample titer ≥3,000 was interpreted as anti-flavivirus or anti-DENV IgG positive.

### Fifty percent focus reduction neutralization test (FRNT_50_)

Macaque sera from each time point were serially diluted and mixed with 40–50 focus-forming units of virus (DENV1 99st12A strain-genotype IV, DENV2 oost22A strain-Asian 2, DENV3-SLMC50 strain-genotype I, DENV4-SLMC318 strain-genotype I and JEV S-982 strain-genotype III). Serum-virus mixture was inoculated to Vero cell monolayer, then 1.25% methylcellulose 4,000 in 2% FCS MEM was added to wells and incubated at 37 °C for 3 days for DENV and 36 h for JEV. The plates were washed with phosphate-buffered saline (PBS) and then fixed with 4% paraformaldehyde solution. Then the cells were permeabilized with 1% NP-40 solution. The plates were blocked with BlockAce for 30 min and treated with 1:1,500 diluted pooled human sera having high anti-flavivirus IgG titers (32) for 1 h at 37 °C. Subsequently, HRP-conjugated goat anti-human IgG (American Qualex, 1:1,500) was added and incubated at 37 °C for 1 h. 0.5 mg/mL of 3,3′-diaminobenzidine tetrahydrochloride (Wako) with 0.03% of H_2_O_2_ solution was added and incubated for 10 min for staining. After washing and air drying, the number of foci per well were counted using a biological microscope. The reciprocal of the endpoint serum dilution that provided 50% or greater reduction in the mean number of foci relative to the control wells that contained no serum was considered to be the FRNT_50_ titer.

### Antibody-dependent DENV infection enhancement assay

FcγR-expressing BHK cell lines were seeded in to a 96 well plate and cultured in Eagle’s minimum essential medium (EMEM) supplemented with 10% heat-inactivated fetal bovine serum (FBS) and G418. Vaccine immunized macaque serum or mouse anti-Flavivirus E monoclonal antibody 4G2 were serially diluted from 1:10 to 1:10,000 with 10% FBS/EMEM, mixed with 30–50 focus-forming units of virus (same DENV strains as those used in FRNT assay) and incubated at 37 °C for 1 h. Virus-immune complex were added to each well of the cells, and cultured for 48 h. The cells were washed with PBS once, dried by air, and fixed with ice-cold methanol-acetone (1:1). After the plates are air-dried, the wells were blocked with 1% horse serum in PBS for 10 min and washed with PBS for three times. The plate was then treated with anti-flavivirus antibody (mAb 4G2 at a dilution of 1:1,000) at 37 °C for 30 min, biotin conjugated anti-mouse IgG antibody (Vector Laboratories, 1:500) at room temperature for 30 min, and avidin-biotin complex (ABC) (Vector Laboratories) solution at room temperature for 30 min. The plates were washed with PBS three times after each incubation step. VIP solution (Vector Laboratories) was added and incubated at room temperature for few minutes until color was developed. Once the plate was washed with PBS, infected cells were quantitated (Keyence BZ-X710 microscope). Fold-enhancement values were calculated using the following formula: (mean infected cells count using FcγR-expressing BHK cells with the addition of mouse serum sample)/(mean plaque count using FcγR-expressing BHK cells in the absence of test sample). Infection enhancement (measured as ADE activity) was tested using serum samples that was diluted from 1:10 to 1:10,000. The fold enhancement values were determined as follows: (the mean number of DENV-infected cells in wells treated with serum samples)/(the mean number of plaques in wells with monolayers incubated in the absence of test samples). The mean value of at least three negative control wells plus three times the SD value was used as the cut-off value to determine which samples had ADE activity.

### Measurement of viremia by RT-qPCR and plaque assay

The level of genomic RNA and the number of infectious virions were determined by RT-qPCR and Plaque Assays as previously described (34). Briefly, marmoset serum was centrifuged at 200 × *g* to remove cells and debris, and viral RNA was extracted (High Pure Viral RNA kit, Roche Diagnostics). 5 µL of viral RNA from each sample was applied to TaqMan RT-PCR using primers and probes specific to the E protein of each DENV serotype (35), and the resulting Cq values were used to calculate the mean viral genomic copies/mL for each marmoset at each timepoint. After calculating the day post-challenge with the highest peak viremia as measured by RT-qPCR, serum samples from the corresponding day were serially diluted 10-fold (from 1:10 to 1:10^6^) in MEM (Sigma-Aldrich) supplemented with 10% FBS. 50 µL of each dilution was added to Vero cell monolayers and incubated for 1 h at 37 °C before removal of infectious media and replacement with MEM supplemented with 2% FBS and 1.25% methylcellulose 3,000. After 72 h incubation, cells were washed with PBS, fixed with 4% paraformaldehyde solution and then stained with crystal violet, and the total number of PFU/mL of sera was calculated for each sample.

### Passive transfer experiment

A total of 15 mL sera drawn at week 0 (pre-immune) or week 12 (4 weeks after final VLP immunization) from the six macaques immunized with the highest dose of DENVLP were pooled together and purified using Melon Gel IgG Spin Purification Kit and NAb Spin column (ThermoFisher #45206, #89961) followed by buffer exchange to PBS via Slide-A-Lyzer dialysis cassettes (ThermoFisher #66012) and concentration to 25 mg/mL via Amicon ultra centrifugal filters (Sigma Aldrich #UFC9100). Purified IgG was sterilized by 0.2 µm filtration before transfer. Eight IFNα/β/γR^-/-^ (AG129) mice aged 4–5 weeks were divided equally into two groups for IP injection of 2.5 mg total IgG from either the pre-immune or VLP-immunized macaques. Mice were then challenged with 1 × 10^6.5^ PFU of DENV2 (strain D2S10) delivered via IP injection at 24 h after passive IgG transfer. All eight mice were then weighed daily until death, extending up to 21 days post-challenge for control animals.

## Data Availability

The raw data used to generate the figures present within this manuscript are available from the corresponding author upon request.

## SUPPLEMENTAL MATERIAL

Supplemental figures jvi.00239-24-s0001.pdf Figures S1 to S3.

ASM does not own the copyrights to Supplemental Material that may be linked to, or accessed through, an article. The authors have granted ASM a non-exclusive, world-wide license to publish the Supplemental Material files. Please contact the corresponding author directly for reuse.
