# Supplementary material for: A tetravalent dengue virus-like particle vaccine induces high levels of neutralizing antibodies and reduces dengue replication in non-human primates
Source: J Virol. 2024 Apr 22;98(5):e00239-24. doi: 10.1128/jvi.00239-24 (PMC11092354; doi:10.1128/jvi.00239-24)
Supplement: Supplemental figures — Figures S1 to S3. [file jvi.00239-24-s0001.pdf]

Supplemental Material

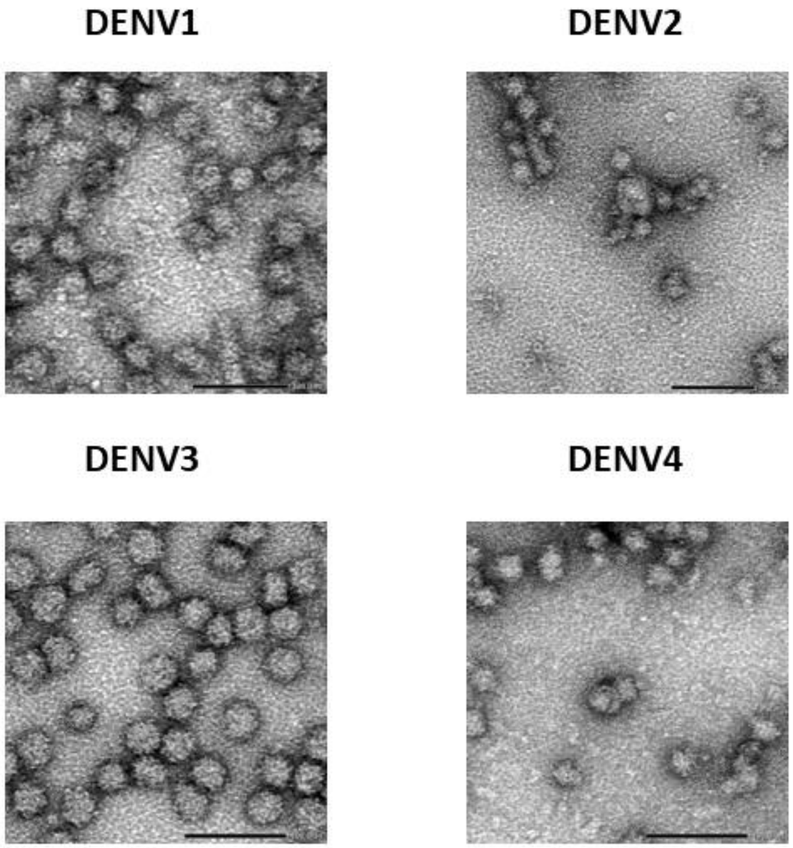

Figure S1. SEM Images of each of the four DENVLPs. The morphologies of DENV VLPs were analyzed by Scanning Electron Microscope at the National Institute of Infectious Disease in Japan (NIID) Microscope Facility to confirm consistency prior to their use in animal studies. Scale bar = 100 nm for all images.

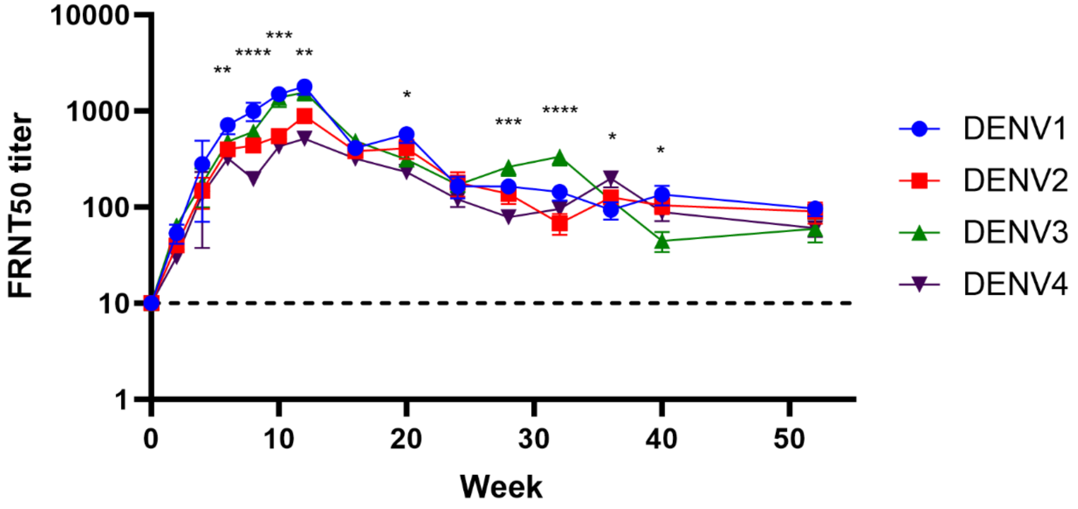

Figure S2. The mean neutralizing antibody titer of all 18 macaques against all four DENV serotypes. The mean neutralizing antibody titer for each serotype and time point as determined by $FRNT_{50}$ assay. Serum samples from each macaque and timepoint were serially diluted and incubated with DENV serotypes 1-4 prior to inoculation of Vero cells. The endpoint dilution capable of neutralizing half of the viral foci relative to Vero cells incubated with DENV1-4 alone was recorded for each macaque and timepoint was recorded, and the mean value for each serotype and sample timepoint was plotted. The statistical difference in mean FRNT50 titer between the four serotypes at each timepoint was measured using a one-way ANOVA with post-hoc Tukey's multiple comparison tests, and the greatest difference between any two serotypes was plotted above each timepoint (* = $p < 0.05$, ** = $p < 0.01$, *** = $p < 0.001$, **** = $p < 0.0001$, unlabeled = $p > 0.05$).

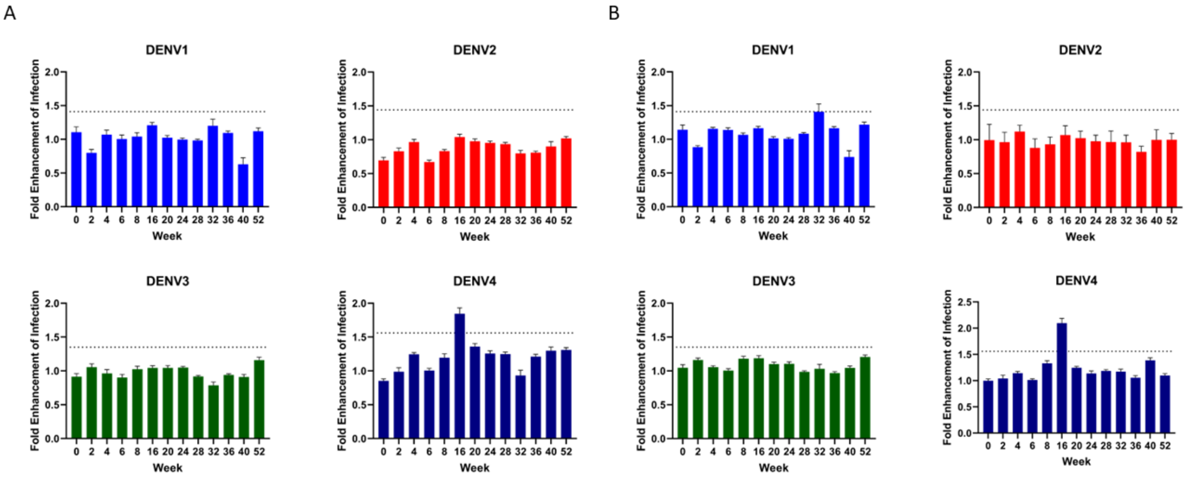

Figure S3. The ADE activity of macaque sera (n=18) at some time points passes enhancement threshold upon further dilutions. **(A+B)** DENV1-4 were incubated with macaque sera diluted 1:100 **(A)** or 1:1000 **(B)** prior to inoculation of FCγR-BHK cells. After two days, infected cells were counted, and the ratio of infected cells in serum- or antibody-incubated wells to infected cells in wells inoculated with virus alone was plotted as the fold enhancement of infection. The mean value plus three standard deviations for three negative control wells was used as the threshold for enhancement (shown as a dotted line in A+B).
